# MAINTENANCE OF HEALTH BEHAVIOR CHANGE: CHALLENGES, IMPLICATIONS, AND FUTURE DIRECTIONS

**DOI:** 10.1093/geroni/igad104.1288

**Published:** 2023-12-21

**Authors:** Jaime Hughes

## Abstract

Health behavior change is critical for healthy aging. Evidence-based programs are available to support a variety of health behavior changes in older adults, including programs for activity, diet, sleep, and medication adherence. Health behavior change programs are typically guided by one or more theoretical models or frameworks describing common constructs related to behavior change. Examples of such constructs include intention, motivation, self-efficacy, and self-regulation. To date, much focus has been on how such constructs relate to initiating and/or achieving behavior change. Although some models specify anticipated outcomes of behavior change over time, maintenance remains largely overlooked. This symposium presents a series of presentations exploring concepts and challenges related to maintenance of health behaviors in older adults. Together, these presentations will explore the definitions and characteristics of maintenance as described in the scientific literature, review maintenance-related trends within physical activity interventions, and discuss findings from stakeholder-engaged sessions designed to identify gaps within the theoretical and scientific literature describing maintenance. The session will conclude with recommendations for future research. This is a Health Behavior Change Interest Group Sponsored Symposium.

